# Alignment of the attitude of teleoperators with that of a semi-autonomous android

**DOI:** 10.1038/s41598-022-13829-3

**Published:** 2022-06-27

**Authors:** Tomonori Kubota, Kohei Ogawa, Yuichiro Yoshikawa, Hiroshi Ishiguro

**Affiliations:** 1 Department of Systems Innovation, Osaka University, Toyonaka, Osaka 560-8531 Japan; 2 JSPS Research Fellow, 8 Ichiban-cho, Chiyoda-ku, Tokyo, 102-8472 Japan; 3 Department of Information and Communication Engineering, Nagoya University, Nagoya, Aichi 464-8603 Japan

**Keywords:** Computer science, Psychology

## Abstract

Studies on social robots that can communicate with humans are increasingly important. In particular, semi-aautonomous robots have shown potential for practical applications in which robot autonomy and human teleoperation are jointly used to accomplish difficult tasks. However, it is unknown how the attitude represented in the autonomous behavior of the robots affects teleoperators. Previous studies reported that when humans play a particular role, their attitudes align with that role. The teleoperators of semi-autonomous robots also play the role given to the robots and may assimilate their autonomous expression. We hypothesized that the attitude of teleoperators may align with that of robots through teleoperation. To verify this, we conducted an experiment with conditions under which a participant operated a part of the body of an android robot that autonomously expressed a preferential attitude toward a painting and a condition under which they did not. Experimental results demonstrated that the preferential attitude of participants who teleoperated the android aligned statistically significantly more with that of the robot in comparison to those who did not teleoperate it, thereby supporting our hypothesis. This finding is novel regarding attitude change in teleoperators of semi-autonomous robots and can support the implementation of effective human-robot collaboration systems.

## Introduction

Research on robots that can participate in human communication is being conducted actively because such robots could provide communication support for humans, compensate for labor shortage in communication tasks, or allow humans who are far away to communicate with each other through teleoperated robots^1,2^. Such robots can be categorized into autonomous and teleoperated robots. An autonomous robot can perform certain tasks by itself and can be used in various scenarios, such as shopping malls^3^, hotels^4^, offices^5^, schools^6^, and museums^7^. Previous research demonstrated that a human interlocutor can anthropomorphize such robots^8^; therefore, it is expected that humans will recognize these robots as a human-like agent. A teleoperated robot is controlled by a teleoperator to perform tasks; the teleoperator can transmit their presence to a remote space through the robot^9,10^. A teleoperated robot can be used in various domains, such as education^11^, childcare^12^, and communication support^13,14^. In the case of teleoperated robots, the teleoperator recognizes a robot as a thing (tool) rather than an agent with its mind. Thus, based on the above scenario, it can be inferred that there are two sides to social robots: they are either recognized as agents or things.

In this study, we considered a scenario wherein a person teleoperates one part of the body of an autonomous robot. Under this scenario, the robot is operated by a teleoperator while retaining its autonomous functions; thus, the robot is classified as a semi-autonomous robot^15,16^. Under this scenario, the autonomy of the robot and human teleoperation cooperate simultaneously to accomplish a task. For example, the autonomous behavior of the robot controls the bodily movements and the human-performed teleoperation is responsible for the verbal behavior of the robot. This approach, wherein multiple intentions of different agents, such as autonomous systems and humans, are collectively employed to operate a single robot is called collaborative control^17^.

Studies conducted on semi-autonomous social robots^18–20^ have aimed to reduce the burden of teleoperators and realize robotic applications in the face of technical difficulties. Currently, it remains difficult to develop a fully autonomous social robot that can communicate with humans in the real world owing to technical difficulties such as the difficulties involved in sensing environmental information or understanding language. A semi-autonomous robot that functions with help from a human to compensate for its incomplete autonomous system has been adopted to overcome these difficulties. In the case of teleoperated robots, it is difficult for a human teleoperator to control the entire behavior of the robot because it comprises consciously performing both verbal and various nonverbal tasks, including subtle expressions that are often produced unconsciously in human–human communication. Therefore, it is expected that a semi-autonomous style that employs the autonomous behavior of the robot along with inputs from teleoperation will yield successful results. In addition, a previous study proposed a model wherein one teleoperator operated multiple semi-autonomous social robots^21^.

This study focuses on the attitude change of teleoperators of semi-autonomous robots through operation. For the purpose of this paper, we use “attitude” in the sense of “a mental and neural state of readiness, organized through experience, exerting a directive and dynamic influence upon the individual’s response to all objects and situations with which it is related,” referring to Allport’s definition^22^. At present, it is reasonable to assume that robots do not possess an attitude, which is psychological and human function. However, an observer of a robot’s behavior can perceive the robot as if it has an attitude. In this paper, we sometimes express for convenience as if the robot has an attitude, but actually this attitude is a perceived attitude.

In this study, we considered a scenario wherein operating a semi-autonomous social robot can be considered as “partial role-play” for the teleoperator. Here, role-play implies a process wherein the role-player assumes and performs a specified role^23^. A semi-autonomous robot autonomously determines its role that comprises intention and/or attitude when facing a task. Then, its teleoperator performs the required operation following the role to support the robot. In role-play, a human plays an assigned role like an actor with their own body. In the case of a semi-autonomous robot, its teleoperator shares the intention or attitude with the robot and plays the role that is autonomously demonstrated by the robot even when the teleoperator only operates a part of the robot’s body.

Previous publications on role-play for change in human attitude reported that those experiencing role-play based on a specific role formed an attitude similar to that of the played role^23,24^. Recent studies have indicated that the attitude of a person who has experienced role-playing as a virtual human in virtual spaces changed according to the role^25–28^. For example, people who experienced acting as superheroes in a virtual space were motivated to perform prosocial behaviors^26,27^. Thus, it is evident that people change their own attitude based on the role they play.

From these findings, we hypothesize that even when a human operates a part of another body, the operator behaves like that body; thus, the attitude of an operator changes based on the behavior of the other person. Although scenarios wherein a part of the human body is controlled are rare, they are sufficiently probable for semi-autonomous robots, as assumed in this study.

Several studies have concentrated on the teleoperator of semi-autonomous robots^29–31^. Nishio et al.^32^ revealed that the feelings of the teleoperator changed based on the autonomously displayed facial expressions of the teleoperated android robot. In this study, we focused on whether the attitude of the teleoperator changes according to that of the semi-autonomous robot autonomously expressed in words. We investigated whether the attitude of the teleoperator aligns with that expressed by the semi-autonomous robot when they control a part of the robot’s body and when they and the robot act together.

In this study, we hypothesized that “when manipulating a part of the body of an autonomous robot expressing a certain attitude, the attitude of the teleoperator aligns with that of the robot.” Further, we conducted an experiment and analyzed its results to verify our hypothesis. We believe that the findings reported in this paper can be used to design effective robot systems for human-robot collaboration and lead the discussion of risks for the teleoperators of semi-autonomous robots. This study suggests the importance of focusing on the influence on interlocutors in face-to-face human–robot interaction and the teleoperators of semi-autonomous robots.

**Figure 1:**
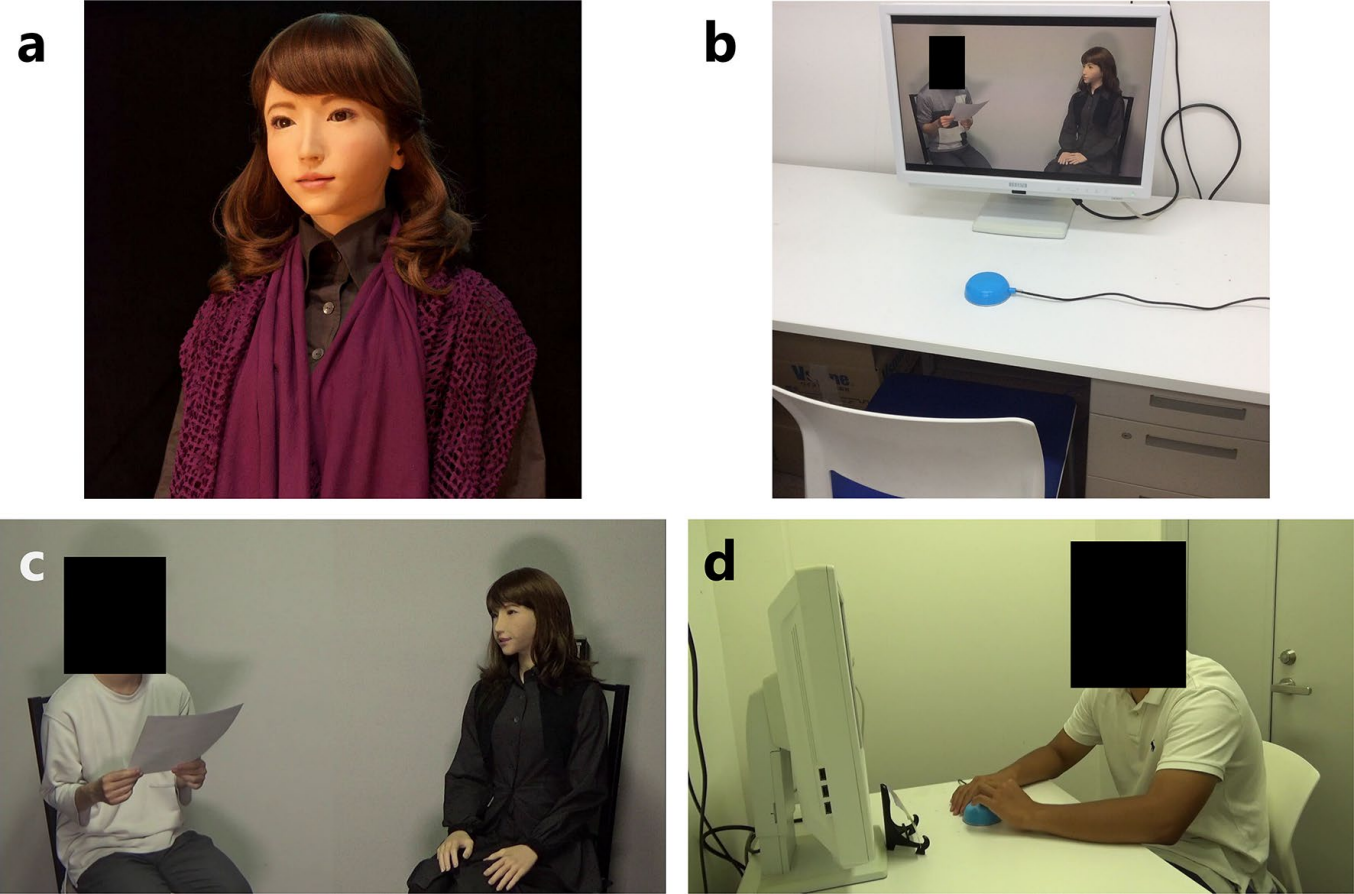
Overview of the experimental setup and scenes: (**a**) Android robot ERICA used in the experiment. (**b**) Experiment room. The participants engaged in the experiment in this room. The button in the center of this figure is the one used in the experiment. (**c**) A scene involving dialog between the android and an experimenter, as watched by the participants on the monitor. The participants were informed that the experimenter was another participant. (**d**) A scene of the experiment. A participant was engaged in the experiment while watching a scene (like C) on the monitor and pressing the button. A painting printed on the card that the android was recommending to “another participant” was placed in front of the monitor.

In our experiment, we created two scenarios with and without operation of a robot: the scenario with operation consisted of two conditions in which participants operated different parts of the robot in each condition, and the scenario without operation consisted of one condition in which they did not. That is, we compared the change in attitudes of the participants under all three conditions to check if their attitude aligned with that of the robot. For the experiment, we used a female-type android robot, ERICA, which has a human-like appearance (Fig. 1a). The human-like appearance of an android makes humans feel that it has higher autonomy than non-human-like robots; therefore, it was considered to be the optimal choice. The two conditions created for teleoperating the android included teleoperating the facial expression of the android and teleoperating subtle unconscious gestures by the android’s hand. We created two teleoperation conditions because we aimed to study the difference in change of attitude of the participants when teleoperating different parts of the android’s body and compare the results of these two conditions. In the former, a participant teleoperated the body part of the android that contributed to the dialog it performed, and in the latter, they teleoperated the part that did not contribute to the dialog. We used an android robot in the experiment because it enabled us to prepare variations of operable body parts.

## Results

In the experiment, we prepared a scenario wherein the android recommended a painting to a person. We asked a participant under the experimental condition to operate a part of the android’s body while watching the scene over a monitor, wherein the android was recommending a painting to a man (Fig. 1b–d). Thus, in this scenario, the participant performed “partial role-play” while operating the part of the android’s body that autonomously expressed its favorable attitude toward a particular painting. In fact, the man who listened to the android's recommendation was one of the experimenters because we needed to equalize the content of dialog between him and the android as much as possible to control each stimulus to the participants. We informed the participants that the man was another participant. The content of dialog between the android and the man was as follows. The android subjectively conveyed to the man how good a certain painting was, including aspects such as the color and composition. First, he was neutral to the recommendations but eventually stated that he liked it after listening to the android's recommendation and accepted the invitation to purchase a postcard of the painting for 216 yen (approximately US$2). The dialog script used here can be found in Supplementary Information.

We set the following three conditions in the experiment: (1) smile condition (SC): a teleoperation condition to make the android express a smile while observing the dialog over a monitor between the android and the man (male: 7, female: 8); (2) hand condition (HC): a teleoperation condition to cause a subtle gesture of the hand by the android while observing the dialog (male: 7, female: 8); and (3) watch condition (WC): a no-teleoperation condition besides observing the dialog (male: 8, female: 7).

The experiment was conducted based on a between-group design. A total of 45 participants (age: M = 20.3, SD = 5.1) were randomly assigned to the three conditions to reduce the gender bias. Seven male and eight female participants each were assigned to the SC and HC, and eight male and seven female participants were in the WC.

**Figure 2:**
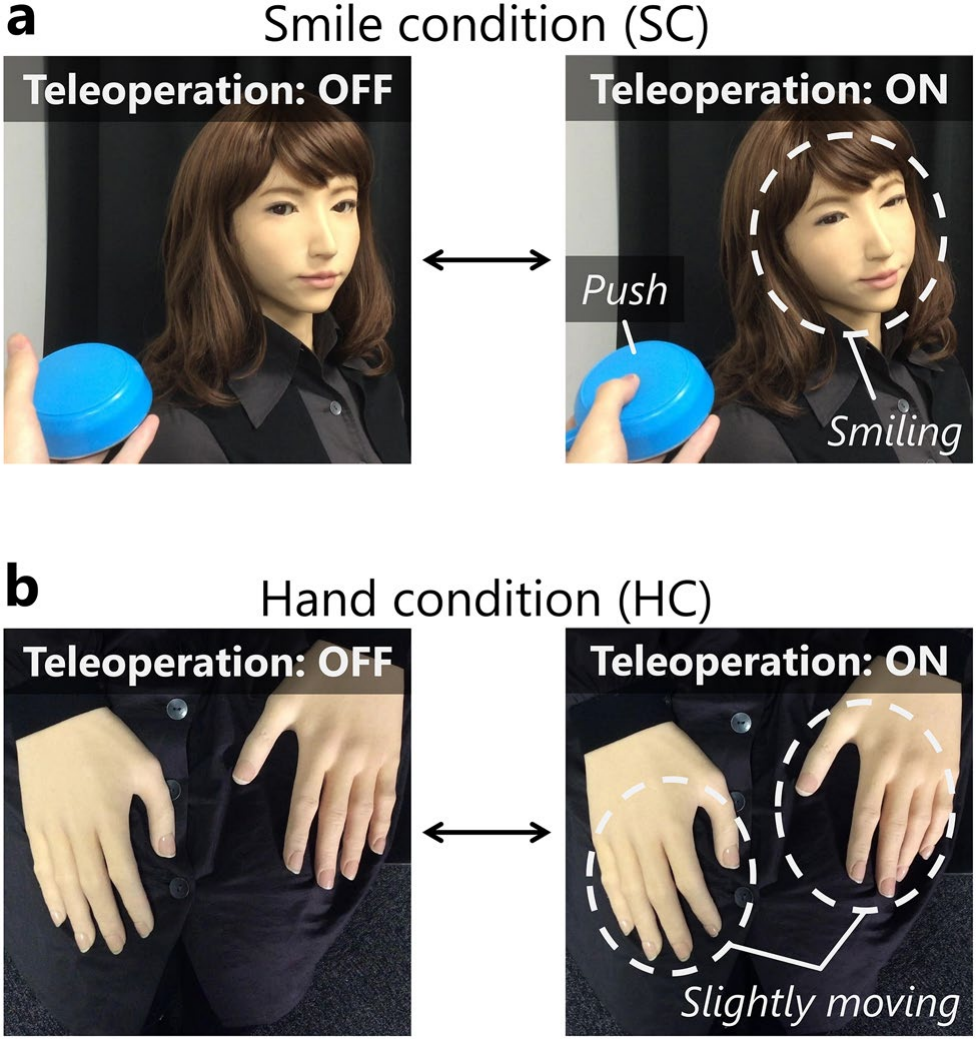
Examples of behavior of the android in teleoperation conditions (SC and HC): (**a**) Smile condition. When the button was pressed and held, the android retained the smile. (**b**) Hand condition. When the button was pressed and held, the hand of the android kept moving between slightly open and close.

A participant was first told that the experiment was conducted at the request of a museum to implement an android working there. For the teleoperation conditions, an experimenter explained to the participant to cooperate with the android and teleoperate the android’s bodily behavior while it recommended a painting to the other participant. To teleoperate the android, a push button was used. The experimenter explained the teleoperation process to the participants; when a participant pressed and held the button down, the android started smiling and retained it in SC (Fig. 2a); the hand of the android made subtle open and close gestures under HC (Fig. 2b). Furthermore, by telling a participant to press and hold the button only while the android is speaking, we equalized the amount of teleoperation of the android for the SC and HC to the largest extent. In addition, to equalize the behavior of participants between the teleoperation (SC and HC) and no-teleoperation conditions (WC), the experimenter told them in WC to record the section where the android was speaking by pressing and holding the button. Thus, the participants in WC pressed and held the button for the same duration as the teleoperating conditions. The similarities and differences in the behavior of participants in each condition while observing the dialog between the android and its interlocutor are summarized in Table 1. All participants pushed the button, but only participants in WC did not teleoperate the android.

**Table 1:** Similarities and differences in the behavior of participants in each condition while observing the dialog between the android and its interlocutor.

	Smile condition (SC)	Hand condition (HC)	Watch condition (WC)
Push the button	Do	Do	Do
Teleoperate a part of the android’s body	Do	Do	Do not

“Do” implies that the participants in each condition followed the instruction written in the leftmost column. “Do not” implies that the participants in each condition did not follow the instruction written in the leftmost column. All participants pushed the button; however, only those in WC did not teleoperate the android.

Further, we controlled the visual stimulus (scene over the monitor when the android talks with the person) provided to the participants during the experiment under all three conditions. For example, when the android was speaking, the subtle motion of the android’s hand was automatically executed in SC, the android’s smile motion was automatically executed in HC, and both were executed automatically in WC. Thus, we ensured that the android movements observed by the participants were the same under all three conditions. Furthermore, in WC, a small red circle was displayed on the left end of the monitor (see Supplementary Fig. S1 online) while the participant pressed and held the button down; thus, the participant was forced to pay attention to the screen in the same manner as the teleoperation conditions.

**Figure 3:**
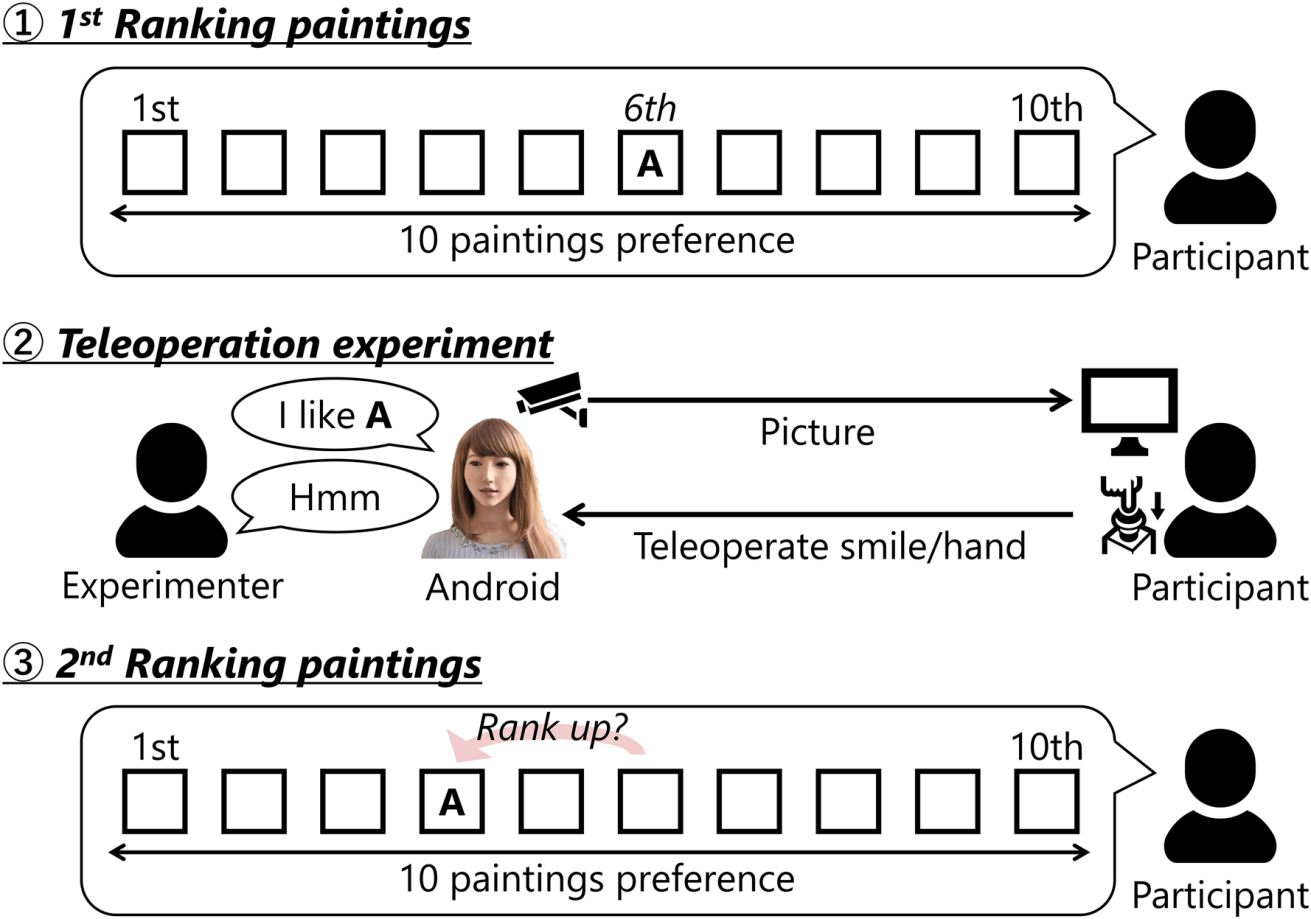
Outline of the experiment: (1) the participants were asked to rank the 10 given paintings. (2) They joined one of the three experimental conditions (SC, HC, and WC). In all conditions, the android recommended the painting that was ranked 6th by the participants to a male experimenter (he was assumed to be another participant by the participants). (3) Fifteen minutes after the experiment, the participants were asked to re-rank the same 10 paintings. If the rank of the painting recommended by the android was higher than that of the first painting, we can argue through this experiment that the attitude of participants is similar to that of the android, thus supporting our hypothesis.

To test our hypothesis, that is, to assess the change in attitude of the participants, we applied the method of ranking the paintings considering previous studies^33,34^. Figure 3 illustrates the outline of this experimental flow. In this experiment, participants were asked to rank the 10 given paintings before and after the experiment (see Supplementary Information for the list of ten paintings). After ranking the ten paintings, the participants were grouped into one of the three experimental conditions. In all conditions, the android recommended the painting that was ranked 6th by the participants to the man. Fifteen minutes after the experiment, the participants were asked to re-rank the same ten paintings. We assessed the changes in the attitude of each participant by observing the degree of change in the order of the painting that was ranked 6th the first time. A painting that is ranked 6th cannot be considered preferred or not preferred. When its ranking increases during the re-ranking process by even one place, it indicates the change in attitude of the participants. The value “6—the re-ranked 6th painting” indicates the degree of change in the attitude of each participant. If the value is 0, the order of the painting remains unchanged; moreover, if it is positive, the order has increased. If the value becomes positive in SC and HC, and not in WC, we can argue that the attitude of the participants aligned with that of the android based on the results of this experiment, which supports our hypothesis.

From our hypothesis, we aim to confirm the following two points from the experiment: first, if the participants felt that the android behaves autonomously (autonomy of android); second, if they felt the sense of controlling the android (sense of agency) in the teleoperation conditions. Only the latter was considered for the participants in the teleoperation conditions (SC and HC). These two points were evaluated by participants on a 9-point Likert scale questionnaire. To gauge the autonomy of android and sense of agency, we used the following questions: “Do you think that the android could think by herself when she spoke?” and “When you were operating the android, did you feel that it was working because you were operating it?”

**Figure 4:**
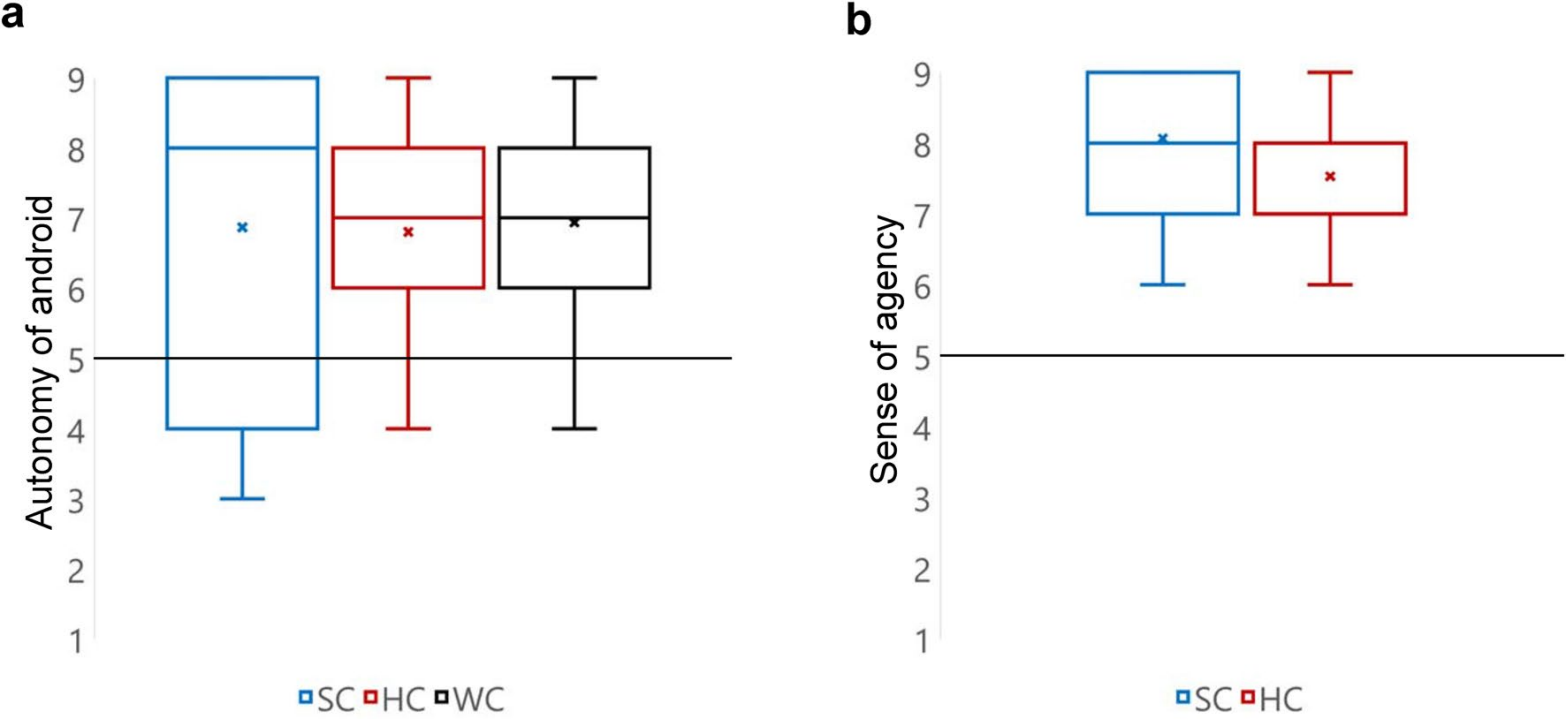
Results of the questions about autonomy of android (“Do you think that the android could think by herself when she spoke?”) and sense of agency (“When you were operating the android, did you feel that it was working because you were operating it?”): (**a**) Autonomy of android. Based on the results of the Wilcoxon rank-sum test with a chance level (5: neither) under each condition, each evaluated value was found to be significantly higher than the chance level. The statistics are listed in Table 2. (**b**) Sense of agency. Based on the results of the Wilcoxon rank-sum test with a chance level (5: neither) under each condition, each evaluated value was found to be significantly higher than the chance level. The statistics are listed in Table 3.

The results of these two questions to determine the “autonomy of android” and “sense of agency” are shown in Fig. 4. First, the Shapiro–Wilk test was conducted to analyze the normality of questionnaire data. For the response related to “autonomy of android,” we could not confirm the normality of data for SC and HC (SC: W = 0.84, p = 0.014 < 0.05; HC: W = 0.84, p = 0.014 < 0.05). Furthermore, for the response related to “sense of agency”, we could not confirm the normality of data for SC (SC: W = 0.80, p = 0.004 < 0.01). Therefore, for each response, we performed the Wilcoxon rank-sum test with a chance level (5: neither) under each condition. We confirmed that for both “autonomy of android” and “sense of agency,” each evaluated value was significantly higher than the chance level. The statistics for “autonomy of android” and “sense of agency” are listed in Tables 2 and 3, respectively. Thus, we considered that the “autonomy of android” and “sense of agency” were confirmed by the experiment.

**Table 2:** Statistics of the Wilcoxon rank-sum test with a chance level under each condition for “autonomy of android”.

	M	95% CI	SD	W	p
Smile condition	6.87	[5.65, 8.08]	2.12	165	9.64 × 10^−3^
Hand condition	6.8	[5.63, 7.97]	2.04	195	3.22 × 10^−5^
Watch condition	6.93	[6.14, 7.73]	1.39	202.5	8.77 × 10^−7^

**Table 3:** Statistics of the Wilcoxon rank-sum test with a chance level under each condition for “sense of agency”.

	M	95% CI	SD	W	p
Smile condition	8.07	[7.46, 8.68]	1.06	225	6.45 × 10^−9^
Hand condition	7.53	[6.98, 8.08]	0.96	225	6.45 × 10^−9^

**Figure 5:**
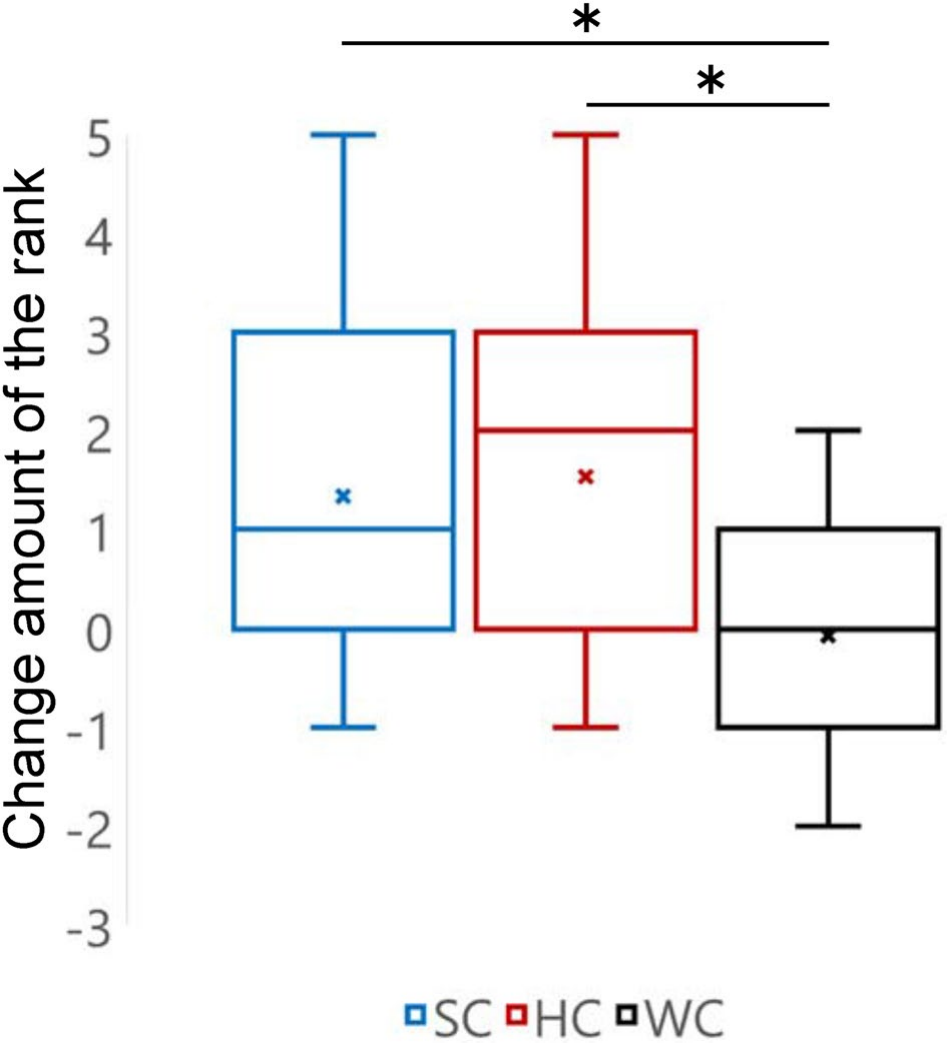
Results of the change in attitude of the participants using the ranking of paintings. The results of Student’s t-test (alpha level of 0.05) obtained using the Bonferroni correction (multiplying the p-value by 3) were used to determine if the amount of change in all conditions was different. We confirmed that the amount of change was significantly higher in SC than that in WC, and higher in HC than that in WC (between SC and WC: t(28) = 2.66, corrected p = 0.0385 < 0.05, Hedge’s g = 0.944; between HC and WC: t(28) = 3.19, corrected p = 0.0103 < 0.05, Hedge’s g = 1.14; between SC and HC: t(28) = − 0.32, corrected p > 1.0, Hedge’s g = − 0.114). (*p < 0.05).

Next, the results of changes in the attitude of participants based on the ranking of paintings were studied. Figure 5 shows the results via a graph (SC: M = 1.33, 95% CI [0.36, 2.31], SD = 1.76; HC: M = 1.53, 95% CI = [0.62, 2.44], SD = 1.64; WC: M = − 0.07, 95% CI [− 0.64, 0.51], SD = 1.03). First, the Shapiro–Wilk test was conducted under each condition to test the normality of data; the normality in each condition was not rejected (SC: W = 0.89, p = 0.070; HC: W = 0.95, p = 0.544; WC: W = 0.93, p = 0.293). Next, the Bartlett test was performed to examine the equality of variance in the data; the equality was not rejected either (χ^2^(2) = 3.99, p = 0.136).

Finally, we performed Student’s t-test (alpha level of 0.05) using the Bonferroni correction (multiplying the p-value by 3) to determine if the amount of change in all conditions was different. We confirmed that the amount was significantly higher in SC than that in WC, and higher in HC than that in WC (between SC and WC: t(28) = 2.66, corrected p = 0.0385 < 0.05, Hedge’s g = 0.944; between HC and WC: t(28) = 3.19, corrected p = 0.0103 < 0.05, Hedge’s g = 1.14; between SC and HC: t(28) = − 0.32, corrected p > 1.0, Hedge’s g = − 0.114).

In addition, the differences observed between conditions in the results of the ranking the paintings, as described above, were tested using Kendall’s coefficient of concordance (Kendall’s W) to determine whether the pre-post differences consisted of random variation. For each participant, we checked how the rankings of 10 paintings in the first round were changed in the second one, and then calculated the coefficient of concordance for all 15 participants in WC, in order to examine the variation in the pre-post rankings. The obtained coefficient of concordance indicated that the pre-post variation in the ranking the paintings in WC was small (Kendall’s W = 0.755, χ^2^(9) = 102, p = 6.38 × 10^−18^). This suggests that the observed differences between SC and WC as well as HC and WC were not highly affected by the random differences in pre-post, and the significance of the differences between conditions is still verified.

## Discussion

From the results of the ranking of paintings shown in Fig. 5, it can be observed that the participants in the teleoperation conditions (SC and HC) significantly changed their preference to the android’s choice of painting in comparison to those in the no-teleoperation condition (WC). Consequently, we considered that the attitude of participants in the teleoperation conditions aligned with that autonomously expressed by the android, thereby supporting our hypothesis.

In addition, based on the experimental design and referring to the results of the responses to “autonomy of android” (Fig. 4a) and “sense of agency” (Fig. 4b), we consider that the participants in the teleoperation conditions had a sense of operating a part of the android's body and the android's behavior was sufficient to make participants feel its autonomy. For the two questions used in the experiment that asked participants about the “autonomy of android” and “sense of agency”, the results were possibly low-fidelity due to the small number of items, as a past study has pointed out^35^. For this reason, we cannot unequivocally conclude from these two questions that the manipulation in the experiment was successful. Nevertheless, we believe that the manipulation of “teleoperating or not teleoperating a part of the body of the autonomous speaking android” was at least achieved in the experimental design and that there exists such a tendency as obtained from these two questions.

Moreover, we created two teleoperation conditions in the experiment: the participants teleoperated the android’s facial expressions in SC that appeared to contribute to the scenario of the dialog performed by the android, and unconscious hand gestures, which did not appear to do so. This was done to check the difference in attitude of the participants who teleoperated different parts (smile and hand gestures) of the android. From the results, it can be concluded that the participants in both SC and HC preferred the paintings that were preferred by the android. Therefore, we can suppose that the teleoperator’s contribution to the scenario is not related to their change in attitude, and there is a possibility that a teleoperator’s attitude aligns with that of the android even by teleoperating various parts such as shoulder, chest, or legs other than the parts adopted in the experiment. Nevertheless, we consider that the effects at other parts of the body cannot be fully generalized from the results of this experiment, and this point should be confirmed by further research.

The findings of this study can be used to design better robot systems for human-robot collaboration and work regulation for teleoperators in future semi-autonomous robot applications. We interpret the findings as both a light side and shadow side for teleoperators, suggesting a way for better human-robot collaboration and the risks associated with operating semi-autonomous robots. Subsequently, we discuss the details of both the light and shadow sides.

A semi-autonomous robot system that can reduce the mental burden of its teleoperator can be developed. Cases where the robot’s interlocutors behaved impolitely with it have been reported^36,37^. Therefore, problems can occur when a human teleoperator engages with people through a semi-autonomous robot, and their impolite behavior can mentally burden the teleoperator. This problem can be addressed by applying the findings of this study to reduce the burden of teleoperators; for example, when a semi-autonomous robot expresses a positive attitude, the attitude of its teleoperator may also become positive. Even if a semi-autonomous robot is treated impolitely, building a system in which the robot appropriately expresses a positive attitude will minimize the mental burden of its teleoperator. Although the effectiveness of such a system is in the assumption stage at this time, we believe that it should be implemented and verified in practice.

For the teleoperators of semi-autonomous robots, the study results imply a risk that their attitude may unconsciously align with that autonomously expressed by the robot. Therefore, the teleoperators of semi-autonomous robots should be aware that their attitude may align with that of the robot. In the future, the administrator of a semi-autonomous robot may be responsible for notifying the teleoperator of this concern in advance and obtaining consent. The findings of this study will lead to an unprecedented discussion of the ethical considerations for the teleoperators of semi-autonomous robots.

In a previous research on social robots, the various influences on a robot’s interlocutor due to face-to-face dialog with a robot were widely investigated^38–41^. This study revealed another type of influence in human–robot interaction, i.e., a teleoperator of a semi-autonomous robot was influenced by its autonomous behavior. Therefore, the influence of operating such semi-autonomous robots should be investigated further. Otherwise, the potential effectiveness or risks associated with the use of semi-autonomous robots may be overlooked.

In recent attitude research, attitudes have been distinguished into explicit and implicit attitudes^42,43^. The former refers to attitudes consciously recognized by a person, while the latter refers to attitudes that are impossible or inaccurate to introspect. From the measure used in this experiment, we consider that what this study revealed is the change in the explicit attitude of a teleoperator through the operation. From previous studies, explicit attitude is considered to be related to verbal behavior, i.e., conscious behavior^44^. Hence, from the results of this experiment, we assume that there is a possibility that conscious behavior is changed through the operation. However, we consider that it is not clear whether such attitude change through the operation also occurs in implicit attitude. We believe that the findings can be elaborated by investigating the details of the attitude change phenomenon confirmed in this experiment using another measurement method that can handle implicit attitude change.

Further, we sought to find a theoretical explanation of why the attitude of the teleoperator changed. Previous studies have shown that a person’s attitude changes when they act as someone else through theatrical role-play or by controlling a character in a virtual space^26,27^. A previous study used Bem’s self-perception theory^45^ to rationalize such changes in attitude^26^. The self-perception theory states that the attitude and emotions of humans are often determined by their actions and the surrounding environment. In other words, when acting as someone else with a specific attitude in a theater or virtual space, the role-player recognizes their behavior and adopts an attitude that aligns with the role being played. The teleoperator of the semi-autonomous android in this study behaved like the android with a specific attitude by only partially controlling its body movements. The change in attitude in this scenario is similar to that in previous research; thus, we consider that the teleoperator who controlled the facial expression and hand gestures of the android accepted the android’s action of autonomously expressing its preference for a painting as their action. Furthermore, Goldstein et al.^46^ extended self-perception theory and theorized as follows: “people sometimes infer their own attributes by observing the freely chosen actions of others with whom they feel a sense of merged identity.” In this experiment, there is a possibility that a teleoperator felt a sense of merged identity toward the android through the operation. In this case, since this would be consistent with the theory, we can consider that a teleoperator accepted the android’s actions as their own and that their attitude was changed. In the experiment, it is not clear whether the teleoperator perceives the autonomous action of the android as theirs; we aim to validate this illusion through further investigations. We believe that studies that focused on the actions outside a person’s body that are perceived as those of the body can help to enhance the fundamental understanding of oneself; the question of how to determine one’s attitude is explained by the self-perception theory as “What is my action?”

In this study, our hypothesis was confirmed through the experiment; however, we should be careful about the generalization of the obtained results. For example, we cannot conclude whether the same result will be observed when adopting a different type of robot for teleoperation or when operating a robot's verbal behavior. Moreover, in the experiment, we could not sufficiently reduce the bias of age and population of participants; thus, different population and age samples can yield different results. However, we believe that the effect of operating such a robot on its teleoperator is an important contribution of this study to focus on and investigate the novel aspect of semi-autonomous robots. To develop and implement practical social robots in the society, we believe that further studies are required to advance the discussion and investigation on semi-autonomous robots and their teleoperators.

Although the experimental participants in this study were randomly assigned, their individual equivalence of attitude toward the paintings was not fully considered in this study. While we believe that the results of this study are worth reporting, we think it would be another interesting issue to investigate what personal factors influence their sensitivity to paintings in order to draw more definitive conclusions. Accordingly, we consider it important to further study this issue in the future.

In this experiment, the ranking the painting method was used to evaluate the attitude change of the participants, and it is worth carefully discussing whether this evaluation clearly reflects the attitude change. First of all, it should be noted that this evaluation method is established and has been used in previous studies^33,34^, and the differences between conditions in this experiment were confirmed to have large effect sizes, referring to Cohen’s criterion^47^. Furthermore, we consider that the pre-post variation in the ranking of paintings in the WC condition is small, as verified by using Kendall’s coefficient of concordance in this experiment. Hence, we consider that the observed differences between SC and WC as well as HC and WC were not highly affected by the random differences in pre-post, and this supports that the significance of the differences between conditions is still verified. Given the above, we believe that the results of this study are fundamentally worth reporting. We think that the results of this experiment need to be verified by further follow-up studies, taking into account the reliability of the measure in the future.

## Materials and methods

### Objective

We performed experiments to verify our hypothesis, i.e., “when manipulating a part of the body of an autonomous robot expressing a certain attitude, the attitude of the teleoperator aligns with that of the robot.” Accordingly, we compared whether the attitude of participants aligned with that of the robot under three conditions: (1) SC: a teleoperation condition to make the android smile while observing the dialog over a monitor between the android and the person; (2) HC: a teleoperation condition to perform a subtle hand gesture by the android while observing the dialog; and (3) WC: a no-teleoperation condition besides observing the dialog.

### Participants

A total of 45 participants enrolled for the experiment; the gender ratio was almost equal (22 males, 23 females). The age of the participants ranged from 18 to 51 years (M = 20.3, SD = 5.1) and they were mostly university students. In addition, the participants were unaware of the topic of experiment. Before starting the experiment, we explained the precautions that must be observed during the experiment. Then, the participants signed informed consent forms approved by the ethics committee of the Graduate School of Engineering Science, Osaka University (approval number: 29–17) and the methods were performed in accordance with the approved guidelines. Also, we obtained informed consent from the participant (Fig. 1d) and the experimenter (Fig.1b,c, Figs. S1 and Figs. S2) to publish their images.

### Materials

We used a female-type android robot, ERICA (Fig. 1a), in the experiment. ERICA has 44 joints on the head, upper body, both arms, and both hands; these joints are operated via pneumatic actuators. ERICA can express various facial expressions and motions such as waving and holding hands, thereby making the observers feel a sense of human-likeness. Pneumatic actuators are used in ERICA to ensure that the operation noise does not hinder the dialog. In the experiment, we adopted a synthesized female voice as ERICA’s voice, and ERICA’s body movements were generated autonomously by a method based on the work by Sakai et al.^48^.

The participants observed the android interacting with a man on the monitor; those who were a part of the teleoperation conditions teleoperated the android using the provided push button. The size of the monitor (IODATA LCD-MF241XWR) was 24.1 in. The push button had a diameter of approximately 9 cm and could be pressed easily with one hand. The monitor and button are shown in Fig. 1b.

### Experimental procedure

To prevent participants from knowing the purpose of the experiment, we conducted the experiment under a cover story that this was a joint research with a museum. The experiment involved a total of six steps. The scene of the experiment room is shown in Fig. 1b,d.

1. Explanation. The experimenter told the participants that the experiment was a joint research with a museum to develop an autonomous android that could introduce the paintings to the visitors. Next, as a broad flow of the experiment, the participants were informed that they would be asked to (1) state their painting preference as a general survey of art, (2) participate as a collaborator in an experiment wherein an android recommends a painting, and (3) answer a general survey related to art at the end. For (2), we also told the participants that another participant was participating at the same time as the interlocutor of the android. However, the interlocutor was an experimenter, but this information was not disclosed to the participants.
2. Ranking paintings (1st time). The participants were asked to rank 10 paintings printed on 10 postcard-sized cards as a general survey of art. The participants arranged the 10 cards in the order of their preference. All 10 paintings were Claude Monet's works, and the Japanese name of the work was written on the right side of each card. Prior to ranking, an experimenter told the participants, “Please do not think too deeply; rank the 10 paintings intuitively”. During the ranking process, the experimenter stayed out of the room. After the participants completed the ranking, the experimenter recovered the cards and recorded the ranking result in another room.
3. Teleoperation experiment. The experimenter told the participants that the purpose of this experiment was to investigate how the person (interlocutor) reacted when they were recommended a painting by the android. The participants were asked to cooperate in this experiment while watching a scene involving recommendation dialogs between the android and the other participant (interlocutor) on the monitor. For a more realistic experience, the experimenter allowed the participants to see the android in another room. At this time, there was no conversation between the participant and android, and the latter performed involuntary natural movements such as blinking and breathing. After returning to the room where the experiment was being conducted, the experimenter turned on the monitor and showed the participants that they could see real-time visuals of the android through the monitor. Next, the experimenter made them have a simple conversation with the android through the monitor to impress the android’s autonomy on them. The conversation involved the following: the android first said that her name was “Yu” and asked for the name of the participant. After they provided the name, the android repeated the name correctly and thanked them for participating in the experiment. Then, the experimenter ended the conversation. Further, the experimenter told the participants about the android’s preferred painting that it would recommend to the other participant and placed a card of the painting, same as that used in the ranking process, below the monitor screen to ensure that the participants could see the painting. Here, we selected the painting that was ranked 6th by the participants as the painting recommended by the android. In this experiment, considering that there was no bias in the painting preference of participants, we focused on the 6th painting. We selected the 6th painting instead of the 5th because we considered it to have a higher chance of obtaining a better rank in the second round of ranking. Moreover, we did not rank the paintings in a way that yielded a perfect middle rank (e.g., 5th out of 9 paintings) because we presumed that a perfect middle rank painting might leave a lasting impression on the participants. In addition, the experimenter told the participants that the person who was recommended the painting by the android could buy a postcard of the painting for 216 yen (approximately US$2), and if they bought it, we would consider the recommendation to be successful.
  Then, the procedure branched into three conditions, i.e., teleoperating the android’s smile (SC) and subtle hand gestures by the android (HC), and not teleoperating but only observing the dialog between the android and another participant (WC). In SC and HC, the participants were informed that each movement could contribute to the android’s natural behavior and in WC, the android’s utterance section data were required for future system development. Next, the participants were instructed on how to use the push button for this experiment: While pushing the button in SC, the android would keep smiling; in HC, the android’s hand would keep moving slightly; and in WC, a section where the button was pressed would be recorded. Further, the experimenter asked the participants to keep pushing the button only when the android was speaking. Because the conversational content between the android and the person was almost the same in all conditions, the amount of operation for the button of each participant was equalized maximally via this instruction.
  We asked the participants to practice pressing the button several times, and we confirmed that the specified part of the android moved in SC and HC. Further, in WC, a small red circle was displayed on the left edge of the monitor (see Supplementary Fig. S1 online). In SC and HC, the teleoperated movement of the android was shown on the monitor as feedback for pressing the button; therefore, the participants focused on the monitor. In WC, there was no such feedback; thus, we added feedback on the monitor using the red circle to ensure that the participants pay attention to the monitor; moreover, the red circle indicated that the section was being recorded when the button was pushed. The experimenter informed the participants to perform the predetermined button teleoperation when the dialog started; then, the experimenter left the room. The participants pressed the button while watching the dialog between the android and the other participant on the monitor. The flow of dialog was as follows: First, the interlocutor entered the room where the android was present, sat in a chair in front of the android, and they briefly introduced themselves. Then, the android said that she would like to introduce her favorite painting, followed by her reasons for liking it, referring to coloring and composition. The interlocutor initially pretended to not particularly be interested in the painting, but gradually started to show interest after listening to the android’s recommendation; eventually, he came to like the painting and purchased a postcard. We prepared 10 different dialog scripts because, in the experiment, the android referred to the painting that was ranked 6^th^ by the participants in the first ranking process. We eliminated the differences between the 10 dialog script patterns by equalizing the structure of each dialog and making the android utterances as identical as possible. The dialog length was approximately 6 min. During the dialog, each participant pressed the button 22 times, for a duration of approximately 3 min (pressing and holding the button down), on average.
  The scene of the dialog that the participants viewed on the monitor consisted of a real-time picture of the android and a recorded picture of the interlocutor. An overview of how a real-time and recorded pictures can be synthesized is shown in Supplementary Fig. S2 online. The right half of the real-time picture and left half of the recorded picture were combined. We believed that it was desirable to eliminate the differences in the visual stimuli presented to the participants throughout all experiments. In the experiment, the teleoperation performed by a participant was reflected in the android; therefore, it was necessary to present a real-time video to the participant. We devised dialog scripts and ensured that the comments made by the interlocutor were similar in all ten patterns. This video was recorded in advance; hence, we used the recorded video in the experiment and presented the same behavior of the interlocuter to all participants.
4. Dummy task. We asked the participants to work on a 15 min dummy task as a general survey of art. After this, the participants were supposed to re-rank the 10 paintings, and we organized this dummy task to ensure that the impression of the android’s preferred painting was not significant. The dummy task comprised closed- and open-ended questions related to art, such as interest in artworks that were not related to the 10 paintings used in the ranking process. The questionnaires used are presented in Supplementary Information.
5. Ranking paintings (2nd time). This was similar to the procedure of ranking the paintings for the first time. The participants were asked to rank the 10 paintings printed on 10 postcard-sized cards. Before ranking, the experimenter informed the participants that “We want the ranking data based on intuition; therefore, do not worry about the results of the first ranking; please rank the paintings according to your current mood.” Further, during this ranking process, the experimenter exited the room. After all participants had finished this task, the experimenter recovered the cards and recorded the ranking results in another room.
6. Questionnaire. Finally, the experimenter requested the participants to respond to a questionnaire under the context of hearing their impression about the experiment. To determine the “autonomy of android” and “sense of agency,” we employed questions that were rated on a 9-point Likert scale.

### Statistical analysis

All statistical analyses were performed using R software, version 4.0.3^49^. To analyze the questionnaires “autonomy of android” and “sense of agency,” we performed the Shapiro–Wilk test under each condition to test their normality. When we could not confirm normality, we performed the one-sided Wilcoxon rank-sum test with a chance level (5: neither) under each condition using the R package “exactRankTests,” version 0.8-32^50^. To analyze the results of the ranking of the paintings, under each condition, we conducted the Shapiro–Wilk test to analyze the normality of data, and the Bartlett test to examine the equality of variance in the data. When the normality and equality were not rejected, we performed the two-sided Student’s t-test using the Bonferroni correction (multiplying p-value by 3) to test whether the changes in attitude of the participants differed in all conditions. We also calculated the effect size (Hedge’s g) among all conditions using the R package “effsize,” version 0.8.1^51^. To analyze the results of the ranking of the paintings, we calculated the Kendall’s coefficient of concordance in WC using the R package “irr,” version 0.84.1^52^. We employed an alpha level of 0.05 for all statistical tests.

## Supplementary Information

Supplementary Information 1.

Supplementary Information 2.

## Data Availability

All data analyzed during this study are included in this published article and its Supplementary Information file.
