# Supplementary material for: Alignment of the attitude of teleoperators with that of a semi-autonomous android
Source: Sci Rep. 2022 Jun 27;12:10473. doi: 10.1038/s41598-022-13829-3 (PMC9237015; doi:10.1038/s41598-022-13829-3)
Supplement: Supplementary file 1 — Supplementary Information 1. [file 41598_2022_13829_MOESM1_ESM.docx]

**Supplementary Information for**

Alignment of the attitude of teleoperators with that of a semi-autonomous android

Tomonori Kubota,^1,2,3,^* Kohei Ogawa,^3^ Yuichiro Yoshikawa,^1^ Hiroshi Ishiguro^1^

**This PDF file includes:**

Supplementary Materials

Supplementary Figure S1, S2

Legend for Supplementary Dataset S1

**Other supplementary materials for this manuscript include the following:**

Supplementary Dataset S1

Supplementary Materials

**Dialog Script used in the Experiment.** We show the dialog script between the android and the man in the experiment. This script has been translated from Japanese to English. There were a total of 10 patterns in the dialog scripts regarding the 10 paintings, and here, we show a version wherein the android recommended “Weeping Willow.” The part enclosed in brackets “[ ]” in the script changed depending on the paintings that the android referred to; however, we made the script as similar as possible for all paintings.

Android Thank you for coming in today. I am Yu, an android.

Man (Bows down to indicate “Thank you.”)

Android Well, may I ask your name?

Man I am *** (man’s name).

Android ***-san. Nice to meet you.

Man Nice to meet you too.

Android By the way, where did you come from?

Man Well, from Suita.

Android Suita? Then, are you a student of Osaka University?

Man Yes, right.

Android I see. Thank you very much.

Man Ah, no problem.

Android So today, I would like to talk a little about paintings. Are you interested in paintings?

Man Well, I do not usually appreciate paintings.

Android I see. I usually introduce paintings in museums, and I would like to introduce some of my recommended paintings to you.

Man I see.

Android Today, I will introduce the painting you were handed. It is called [“Weeping willow”]. Have you seen this painting before?

Man No.

Android Is that so. This is my favorite painting! Please look at it. How about that?

Man Hmm. Well, it feels normal.

Android I see. I like the colors used in [“Weeping Willow”]. I prefer [a slightly dark image and a calm feeling]. Do you like this type of color scheme?

Man Hmm... Color... I am not sure; I cannot say which.

Android Is that so. How about the expression of light? [Even in dark colors, the effect of light is expressed]. How about that?

Man Ummm... I feel yes and no.

Android I see. ***-san, is there any part of this picture that interests you?

Man Let me see... I do not know how to say it, but is it called balance? I feel the balance is good.

Android Indeed. Certainly, I also think the balance of [“Weeping Willow”] is good. The composition is that [there is a tree in the middle and the space is divided appropriately into the left and the right].

Man I agree. I feel it is simple.

Android Yes. It is simple, but does it look cool?

Man Yes, I feel the composition is cool, somehow.

Android I agree. I also like the theme of [“Weeping Willow”]. Does not seem like anything, but when it is drawn as a painting, it is so impressive. Do you not feel that way?

Man Oh, I see. Indeed.

Android Right. This painting is interesting because I have seen it many times and discovered many things.

Man Oh yeah? It sounds a little interesting when you say so.

Android That is right. How is it? Do you like my favorite work, [“Weeping Willow”]?

Man Yes. I like it a lot more than when I first saw it.

Android That was good. Today, the postcard of [“Weeping Willow”] is being sold for 216 yen. If you like it, how about it?

Man Really. ...Hmm, then, I would like one.

Android Thank you very much. Please let the staff know after the experiment is over.

Man I see.

Android I am glad that you like the picture.

Man Yeah.

Android Well, our talk is over this time, thank you very much.

Man Thank you very much.

Man (Leaves the room)

**The List of Ten Paintings used in the Experiment.** All ten paintings were drawn by Claude Monet. Owing to copyright restrictions, we have not shown the images of the paintings.

- Apple trees in blossom (1873)
- Boulevard des Capucines (1873)
- Charing Cross Bridge (1903)
- Landscape: The Parc Monceau (1876)
- The Saint-Lazare Station (1877)
- Marine View with a Sunset (1875)
- The Tuileries (1876)
- Vétheuil (1879)
- Waterloo Bridge (1903)
- Weeping Willow (1918-1919)

**Questionnaires used in the dummy task.** In the dummy task, the participants answered the following seven mandatory closed-ended questions and five non-mandatory open-ended questions. The participants were asked to work on the dummy task for the full 15 minutes; however, it was not necessary to answer all the open-ended questions.

Closed-ended questions: these were 9-point Likert scale questions (1: Not at all – 9: Very much).

- I am interested in painting.
- I am interested in various kinds of art, not just paintings.
- I often go to museums.
- My friends often go to museums.
- I would like to visit museums as much as possible if I have the chance.
- I prefer contemporary art to classical art.
- I prefer Eastern art to Western art.

Open-ended questions: For the first question given below, we used three paintings that were considered to be different in style from Monet’s paintings used in the experiment in order not to influence the participants’ preferences in the ranking painting task. Owing to copyright restrictions, we have not shown the images of the three paintings herein.

- Please write freely what you think is good or not good about the following paintings. Also, if you were to recommend this painting to someone else, how would you recommend it?
  - Félix Vallotton, “High Alps, Glaciers and Snowy Summits” (1919)
  - Piet Mondrian, “Gray Tree” (1911)
  - Félix Vallotton, “The Visit” (1899)
- What do you think is the most effective way to communicate the appeal of painting and art to college students? You can use bullet points, so feel free to write them.
- Please freely think of a museum or exhibition that you think would make you want to visit and tell us about it.
- If you were to combine robots or artificial intelligence with art, what do you think would be interesting?

Supplementary Figures


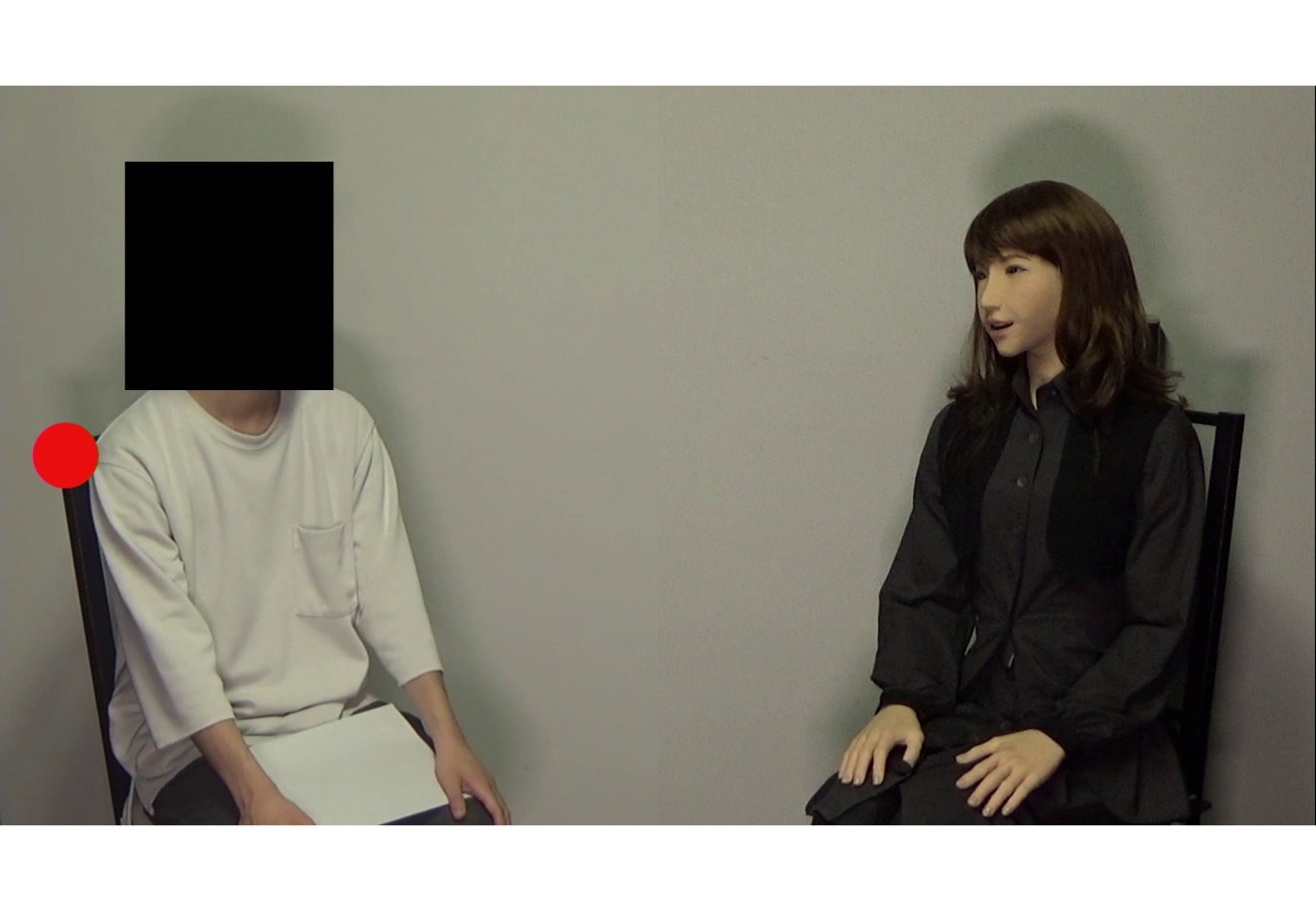


**Figure S1. Scene of the dialog between the android and an experimenter, as watched by participants in WC.** A small red circle was displayed on the left side of the scene while a participant kept pressing the button.


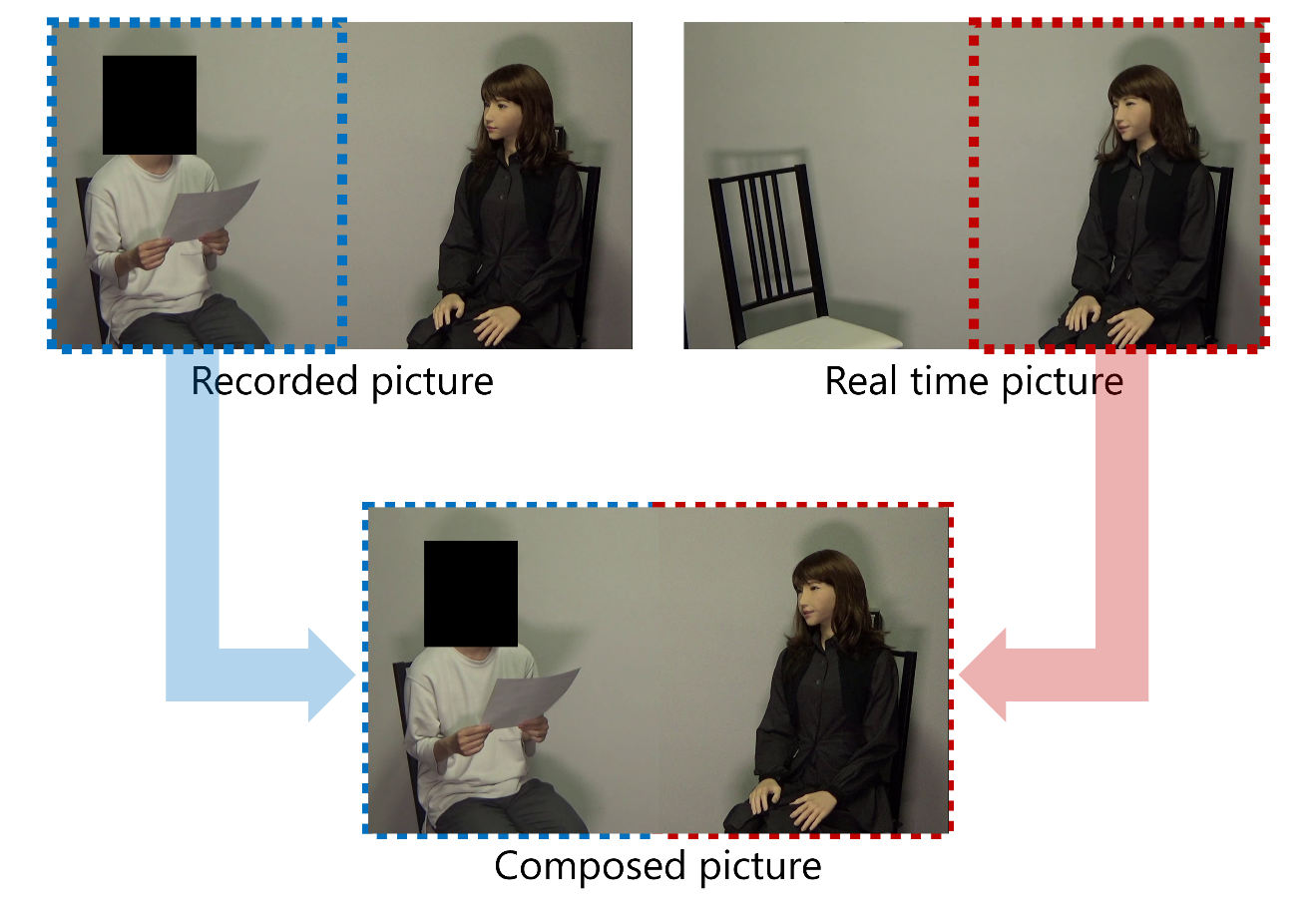


**Figure S2. Outline of how to synthesize the real-time and recorded pictures.** The right half of the real-time picture and left half of the recorded picture were combined.

Supplementary Dataset S1 (separate file). The original raw data of the experiment. The first row mentions the condition, the second row shows the result of “autonomy of android,” the third row shows the “sense of agency,” and the fourth row is the degree of change in attitude determined by using the ranking of the paintings (6 - the re-ranked 6th painting).
